# Nutritional Value of *Nannochloropsis oceanica* for Weaner Piglets

**DOI:** 10.3390/ani14243575

**Published:** 2024-12-11

**Authors:** Andreia A. M. Chaves, David M. Ribeiro, Cátia F. Martins, Tatiane Fernandes, Margarida R. G. Maia, António J. M. Fonseca, Ana R. J. Cabrita, Susana P. Alves, Mário Pinho, Rui J. B. Bessa, André M. de Almeida, João P. B. Freire

**Affiliations:** 1 LEAF—Linking Landscape, Environment, Agriculture and Food Research Center, Instituto Superior de Agronomia, Universidade de Lisboa, Tapada da Ajuda, 1349-017 Lisboa, Portugal; andreiachaves@isa.ulisboa.pt (A.A.M.C.); davidribeiro@isa.ulisboa.pt (D.M.R.); catiamartins@isa.ulisboa.pt (C.F.M.); mmaia@isa.ulisboa.pt (M.R.G.M.); aalmeida@isa.ulisboa.pt (A.M.d.A.); jpfreire@isa.ulisboa.pt (J.P.B.F.); 2 Associate Laboratory TERRA, Instituto Superior de Agronomia, Universidade de Lisboa, Tapada da Ajuda, 1349-017 Lisboa, Portugal; 3 CIISA—Centro de Investigação Interdisciplinar em Sanidade Animal, Faculdade de Medicina Veterinária, Universidade de Lisboa, Avenida da Universidade Técnica, 1300-477 Lisboa, Portugal; fernandest@vt.edu (T.F.); susanaalves@fmv.ulisboa.pt (S.P.A.); 4 REQUIMTE, LAQV, ICBAS, School of Medicine and Biomedical Sciences, University of Porto, Rua Jorge Viterbo Ferreira, 228, 4050-313 Porto, Portugal; arcabrita@icbas.up.pt (A.R.J.C.); mpinho@fmv.ulisboa.pt (M.P.); 5 AL4AnimalS—Associate Laboratory for Animal and Veterinary Sciences, Avenida da Universidade Técnica, 1300-477 Lisboa, Portugal

**Keywords:** apparent total tract digestibility, digestible energy, feed evaluation, microalgae, post-weaning piglets

## Abstract

This study evaluates the nutritional value of spray-dried *Nannochloropsis oceanica* biomass to infer its applicability for weaner piglet feeding. Twenty-four piglets were randomly allocated to four dietary treatments (*n* = 6) with increasing *N. oceanica* inclusion (0, 5, 10, and 15%) levels. After a 4-day adaptation period, the digestibility experiment lasted two weeks, with daily excreta collection. Total tract apparent digestibility (TTAD), N balance, intestinal pH, histomorphology, and volatile fatty acid (VFA) concentrations were measured. No significant differences were observed in feed intake, growth, or intestinal morphology (*p* > 0.05). N intake increased linearly (*p* = 0.005) with *N. oceanica* inclusion, while N retention efficiency remained unchanged (*p* = 0.058). VFA concentrations decreased linearly (*p* < 0.001). The TTAD of dry matter (DM), organic matter, ether extract, and gross energy decreased (*p* < 0.05) with higher *N. oceanica* levels, while the ash TTAD increased (*p* < 0.001). The estimated TTAD values for *N. oceanica* biomass were 72.5% for DM, 82.9% for N, and 64.4% for gross energy. The digestible energy, metabolisable energy, and digestible crude protein values estimated for *N. oceanica* were 12.74 MJ/kg DM, 12.40 MJ/kg DM, and 20.4% DM, respectively. This information enabled the first nutritional characterisation of *N. oceanica* for weaner piglets, ultimately leveraging its use in piglet diet formulation.

## Simple Summary

Novel sustainable alternative protein sources for animal feeding are needed. Microalgae are a promising option due to their interesting high protein content. However, information regarding their nutritional value remains scarce, and until now, a detailed nutritional evaluation of *Nannochloropsis oceanica* for swine has not been reported. This study fills this gap by determining the apparent digestibility, digestible and metabolisable energy, and nutritional value of *N. oceanica* when included in the diets of weaner piglets allowing its use as a feed ingredient in commercial dietary formulations.

## 1. Introduction

The growth of the global human population, which will reach 10 billion by 2100, will lead to an increase in the production and consumption of animal products, mainly pork and chicken meat [1]. This will imply a higher demand for conventional feedstuffs such as corn or soybean meal, widely used in both livestock and human nutrition [2]. The production of such crops is associated with several negative environmental impacts, such as high water, fertilisers, and arable land use, land use ultimately contributing to habitat loss, and greenhouse gas emissions linked to the international commerce of such commodities [3,4]. It is therefore of the utmost importance to establish novel and sustainable alternative feedstuffs for monogastric feeding. Recently, several studies have been conducted on alternatives to conventional feedstuffs, and microalgae are among the most promising [5].

Microalgae are unicellular photosynthetic organisms that, depending on the species, strain, and production conditions, show a wide variability in their protein, lipid, carbohydrate, vitamin, and mineral contents [5]. Moreover, their protein content is, in many cases, similar to or even higher than those of conventional feedstuffs commonly used in animal feeding [6]. Nevertheless, large differences in microalgae nutrients and energy content are reported reflecting the high number of species and production conditions. In addition, the use of microalgae in animal nutrition is currently limited by the high manufacturing costs and composition heterogeneity of each batch produced, even under similar production conditions [4]. Among eukaryotic species, interest has emerged in the *Nannochloropsis* genus.

*Nannochloropsis* spp. are green eukaryotic microalgae with higher growth rates when compared to other genera [7]. Their biomass has been described to have 267–430 g/kg crude protein (CP), 96–359 g/kg carbohydrates, and 153–300 g/kg of total lipid content on a dry matter basis (DM), as reviewed by Zanella and Vianello [8]. *Nannochloropsis* spp. also have high concentrations of n-3 polyunsaturated fatty acids (PUFAs), particularly eicosapentaenoic acid (EPA), which might modulate the animal’s immune response [9], further increasing the interest in its use. Vitamins and pigments produced by these microalgae also significantly contribute to their interesting nutritional value [8]. However, the availability of such valuable nutrients may be limited by the cell wall of *Nannochloropsis* spp. which has been described to be particularly difficult to digest [7].

The weaning period is the most stressful event in the pig’s productive life. This is due to social, environmental, physiological, and nutritional changes. During this period, the piglet’s gastrointestinal tract must adjust to digest new feedstuffs to which it is not yet fully adapted [10]. This can lead to digestive problems, inflammatory complications, nutrient absorption problems, and/or diarrhoea [11]. Further challenges may be presented by feeding alternative feedstuffs such as microalgae. Indeed, dietary microalgae supplementation might result in digestive disturbances, likely caused by amino acid imbalances, a reduction in the buffering capacity of the gastrointestinal tract, and electrolyte imbalances caused by its high ash content [6]. For this reason, the feed provided at this stage must be of high quality, reducing potential health problems and promoting healthy growth.

We hypothesise that *Nannochloropsis oceanica*, due to its n-3 PUFA and protein contents [12], will be well digested by weaner piglets and will thus be a suitable feedstuff for such animals when its price becomes competitive. Therefore, we have evaluated the digestibility and energy value of *N. oceanica* for weaners. In fact, and to the best of our knowledge, the nutritional values herein presented for spray-dried *N. oceanica* for swine are the first to be reported. This study will significantly contribute to assessment of the applicability of this microalga as a feedstuff for piglet diets, particularly during the weaner phase.

## 2. Materials and Methods

### 2.1. Animals and Experimental Diets

The experimental trial was conducted at the Animal Production experimental facilities of the School of Agriculture (Instituto Superior de Agronomia—ISA) at the University of Lisbon, Lisbon (Portugal). All procedures were approved by the ISA Animal Experimentation Committee and the Animal Care Committee of the Portuguese National Veterinary Authority (process number 0421/000/000/2019), in compliance with national and European Union legislation (2010/63/EU Directive). Twenty-four male piglets (Pietrain × (Large White × Landrace)) weaned at 28 days of age were purchased from a commercial farm and transferred to the ISA facilities two weeks later.

Piglets had an initial body weight of 15.5 ± 1.37 kg (mean ± SD) and were individually housed in metabolic cages (1000 × 500 × 480 mm), with an infrared heating lamp and water available ad libitum as previously described [13]. The animals were randomly allocated to one of four dietary treatments (*n* = 6): control (wheat-, corn-, and soybean meal-based diet), NCO5% (95% control diet and 5% *N. oceanica*), NCO10% (90% control diet and 10% *N. oceanica*), and NCO15% (85% control diet and 15% *N. oceanica*). The stepwise incorporation of the microalga was conducted by replacing the whole basal diet to estimate the microalga nutritional parameters by regression. The microalga *N. oceanica* used in this experimental trial was produced locally autotrophically, in photobioreactors, supplied as a feed-grade spray-dried powder (particle size < 40 μm) by Allmicroalgae—Natural Products SA (Pataias, Portugal) and then incorporated into the diets. The composition of the experimental diets and microalgae is described in Table 1 and Table 2, respectively. After an adaptation period of 4 days, the sampling period lasted 2 weeks. The experimental diets were fed to the piglets in the form of pellets during the adaptation period and the sampling period.

### 2.2. Animal Performance, Faecal Score, and Urine and Faeces Collection

During the experiment, feed intake was equalised and controlled between groups through restring feeding, as this was the only way to strictly control the feed consumption, accurately determining digestibility. In the first week of the trial, each group was fed with 5.96 kg (as-fed basis) of the diet, which was increased to 6.6 kg (as-fed basis) in the second week. Piglets were weighed weekly, on days, 0, 7, and 14, before feeding. To assess the total tract apparent digestibility (TTAD) and the nitrogen balance of these diets throughout the 14 days of the experiment, feed intake, refusals, faeces, and urine were collected, weighed, and recorded daily from each animal. The refusals, faeces, and urine were stored daily at −20 °C. Regarding urine samples, 50 mL of sulphuric acid (5 vol.%) was added daily to the urine collection recipients to prevent nitrogen loss. At the end of the trial, for each animal and per week, a composite faecal and urine sample was preserved until further analysis. Daily DM intake was determined by calculating the difference between the DM of the feed provided and the DM of the leftovers over time. The average daily gain (ADG) was calculated by determining the difference between the final weight and the initial weight over the specified period.

### 2.3. Slaughtering and Sampling

At the end of the trial, piglets were slaughtered by exsanguination with previous electrical stunning and eviscerated following applicable legislation. The contents from the stomach, duodenum plus jejunum, ileum, caecum and colon were collected and immediately analysed for viscosity and pH determination. The contents of the caecum and colon were also stored at −20 °C for volatile fatty acid determination. Three sections of the small intestine were collected for histological analysis: the duodenum (10 cm below pylorus), jejunum (5.5 m below pylorus), and ileum (60 cm above ileum–caecal valve). The tissues were placed into a 10% buffered formalin solution and then processed for paraffin embedding.

### 2.4. Analysis of Faeces, Diets, and Microalgae

Composite faecal samples were dried at 60 °C for 72 h, in an oven with ventilation. Dried faeces and diets were ground with a 1 mm diameter mesh mill (SK100 comfort miller, Retsch, Haan, Germany). Faeces, diets, and microalgae samples were analysed for DM, ash, CP, ether extract (EE), and energy contents following AOAC methods [15]. The DM content was obtained by oven-drying at 103 °C to a constant weight. The ash content was determined following incineration at 550 °C (method 942.05). The nitrogen content was determined using the Kjeldahl method (method 954.01), and the CP content calculated as N × 6.25 for diets and faeces, and as N × 4.78 for microalgae [14]. The EE was determined, after acid-hydrolysis pre-treatment, by automatic Soxhlet extraction with petroleum ether [15] (method 920.39). The NDF (with heat-stable amylase and without sodium sulphite) and ADF contents of faeces, diets, and microalgae were determined sequentially according to Van Soest et al. [16], and expressed inclusive of residual ash. The lignin content of the diets was also determined [17]. Due to the small particle size of the microalgae, the filtration step used for its NDF and ADF content quantification was performed with a glass microfiber filter, as described by Cabrita et al. [18]. The energy contents were determined by complete sample combustion using a PARR 1261 calorimeter (Parr Instrument Company, Moline, IL, USA). The fatty acid methyl esters of the faeces, diets, and microalgae were prepared by direct transesterification and analysed by gas chromatography with flame ionisation detection (GC-FID), as described by Alves et al. [19]. Briefly, samples previously freeze-dried using a CoolSafe Superior Touch 95 freeze-dryer (Labogene, Alleroed, Denmark) at −92 °C and 0.2 hPa, were transesterified with sodium methoxide in methanol at 50 °C followed by transesterification with hydrochloric acid in methanol at 80 °C. Before transesterification, 1 mL of internal standard (methyl nonadecanoate at 1 mg/mL) was added. The chromatograph used was a Shimadzu GC-2010 Plus (Shimadzu Corp., Kyoto, Japan) equipped with an SP-2560 capillary column (100 m, 0.25 mm i.d., and 0.20 μm film thickness; Supelco Inc., Bellefonte, PA, USA). The chromatographic conditions were as follows: injector and detector temperatures were maintained at 220 °C and 250 °C, respectively; an initial oven temperature of 50 °C was held for 1 min, increased at 50 °C/min to 175 °C and held for 35 min, and then increased at 2 °C/min to 220 °C and held for 40 min. Helium was used as the carrier gas at a flow rate of 1.28 mL/min, and 1 μL of sample was injected with a split ratio of 1:50.

The mineral content of the microalgae was determined using inductively coupled plasma–optical emission spectrometry according to the method described by Ribeiro et al. [20]. This analysis was performed using an iCAP 7000 Series, ICP-OES spectrometer (Thermo Scientific, Waltham, MA, USA), equipped with an automated sampler.

The analyses were performed in duplicate for faecal samples and in triplicate for the diets and microalgae.

### 2.5. Urine Samples Analysis

The nitrogen content of the composite urine samples was analysed using the Kjeldahl method, as described by AOAC [15]. This procedure was performed after thawing the pooled urine samples. Then, urine samples were freeze-dried using a CoolSafe Superior Touch 95 freeze-dryer (Labogene, Alleroed, Denmark) at −92 °C and 0.2 hPa, for 5 days, followed by the complete combustion of each sample using the previously mentioned calorimeter. The analyses were performed in duplicate per urine sample.

### 2.6. Viscosity Determination

Samples of the small intestine contents were centrifuged at 18,144× *g* for 10 min in a J2-HS ultracentrifuge (Beckman-Coulter, Brea, CA, USA), and the supernatant viscosity was measured in duplicate, using an LVDVCP-II viscometer (Brookfield Engineering Laboratories, Middleboro, MA, USA) set to 6 rpm at 23 °C. Procedures were conducted as described by Martins et al. [21].

### 2.7. pH Measurements

The stomach, duodenum with jejunum, ileum, caecum, and colon contents’ pH values were determined immediately upon collection, using a glass electrode pH meter (Metrohm 744, Metrohm AG, Herisau, Switzerland).

### 2.8. Histological Analysis

Tissue samples (7 μm thick) were stained with haematoxylin–eosin and examined microscopically to measure the heights and widths of the villi, as well as the depths of the crypts, as described by Martins et al. [21]. This procedure was carried out using a BX 51 microscope (Olympus, Tokyo, Japan), equipped with 4× and 10× objectives, and images were captured digitally with a DP 21 camera (Olympus, Tokyo, Japan). The dimensions of the villi and the depth of the crypts were analysed using Olympus DP-Soft software 3.0 (Olympus, Tokyo, Japan). For each animal, ten intact and properly oriented villi and crypts were selected from each intestinal region.

### 2.9. Volatile Fatty Acids Determinations

Samples of cecum and colon contents collected for volatile fatty acid determination were stored in 4 mL of orthophosphoric acid solution 5% (*v*/*v*) at −20 °C. These samples were then analysed and quantified as described by Oliveira et al. [22] using 1 mL of sample. Briefly, 130 µL of internal standard (iso-6:0 at 50 mmol/L) was added to previously stored samples, centrifuged (15,000× *g* for 15 min), and then analysed using the chromatograph Shimadzu 2010Plus (Shimadzu, Kyoto, Japan) equipped with a flame ionisation detector and a fused capillary column (Nukol, 30 m × 0.25 mm i.d. × 0.25 μm film thickness, Supelco Inc., Bellefonte, PA, USA).

### 2.10. Statistical Analysis

Data were checked for the normal distribution and homogeneity of variance of the residues. For the analysis of TTAD and nitrogen balance data, the week of the sampling period (Wk) and the diet (D) were included in the model as fixed effects, and the repeated measurements within the piglets were accommodated in the model using the unstructured covariance structure. The interaction between the week of measurements and the diet was never significant (*p* > 0.05, Table S1). For the remaining variables, the model considered only the dietary treatment as the single fixed effect. Polynomial contrasts were used to test the linear and quadratic effects resulting from the dietary incorporation of increasing levels of microalgae. Significance was declared at *p* < 0.05.

For variables that showed no linear effect of *N. oceanica* incorporation on diets’ TTAD, the estimates of the biomass TTAD presented were the common LSMeans, SEM, and confidence limits of the three dietary treatments containing the microalgae obtained through the “estimate” statement of Proc MIXED.

When a linear effect of dietary *N. oceanica* incorporation on the TTAD of the nutrients in diets was observed, a mixed model was fitted that considered the proportion of *N. oceanica* incorporated into the diet as a continuous variable, the week of measurements as a fixed effect, and the animal as a random factor. The TTAD for *N. oceanica* biomass was estimated by solving the regression model for 100% *N. oceanica* incorporation.

## 3. Results

### 3.1. Feed Intake, Weight Gain, and Faecal Dry Matter

Table 3 presents data on the feed intake, weight gain, and faecal dry matter content. The diets did not affect these variables (*p* > 0.05). Across treatments, the final live weight, intake, and ADG averaged 23.2 kg, 843 g/d, and 550 g, respectively. The faecal DM content showed no differences between the groups (*p* > 0.05, Table 3) and was higher in the first than in the second week of measurements (321 g/kg vs. 261 g/kg), but neither the effects of the diet nor of the week × diet interaction on the faecal DM content were significant (*p* < 0.05, Table S1).

### 3.2. Total Tract Apparent Digestibility, Nitrogen Retention, and Energy Values of Diets

The effects of *N. oceanica* inclusion in piglet diets on TTAD, nitrogen balance, digestible energy (DE), and metabolisable energy (ME) are presented in Table 4 and Table 5. Increasing levels of *N. oceanica* dietary inclusion had a linear effect (*p* < 0.05) for all the nutrients’ TTAD variables, except for N (*p* = 0.15). The TTAD of DM, OM, EE, and gross energy (GE) decreased linearly (*p* < 0.05) with increasing *N. oceanica* dietary incorporation, whereas the ash TTAD increased linearly (*p* < 0.05). Only the TTAD of NDF and ADF displayed a quadratic response (*p* < 0.05), with NDF increasing up to NCO10%, and the TTAD of ADF being maintained at NCO5% and decreasing sharply after that inclusion level.

The fatty acid TTAD values were mostly above 90%, except for 16:0 and 18:0. The TTAD value of 18:0 was quite low, ranging from 3.5 to 20%. Increasing the dietary inclusion of *N. oceanica* had no effect (*p* > 0.05) on the TTAD of 16:0, 16:1c9, and 18:0 but linearly decreased (*p* < 0.03) the TTAD of 18:1c9, 18:2n-6, and 18:3n-3. The control diet did not contain 20:4 n-6 and 20:5n-3; thus, the effects on its TTAD were evaluated only on dietary treatments containing microalgae. No impact of the microalgae’s increasing incorporation was observed for the TTAD of 20:4 n-6 and 20:5 n-3 which averaged 91.7 ± 0.33% and 94.2 ± 0.24% across treatments, respectively.

The N intake and retention (expressed as g/d) increased linearly (*p* < 0.05) with *N. oceanica* dietary inclusion. The proportion of ingested N that was retained (i.e., N practical retention coefficient—NPRC) did not differ (*p* > 0.05) between diets and averaged 641. Similarly, the proportion of N absorbed that was retained (i.e., N retention coefficient—NRC) also did not differ (*p* > 0.05) between diets, averaging 771.

The DE and ME contents of diets showed a quadratic response (*p* < 0.05), decreasing until 10% *N. oceanica* incorporation and remaining stable (for DE) or increased (for ME) for 15% *N. oceanica* incorporation. The ME/DE ratio was not affected by the diet (*p* > 0.05) and averaged 0.972.

All of the TTAD and energy values determined were affected by the week (Table S1), with lower (*p* < 0.05) TTAD and energy values in the second week of the trial. Thus, the TTAD of DM and ME values in the second week were 2.5% and 2.9% lower, respectively, than in the first week of the trial. No week × diet interactions were detected.

### 3.3. Estimates of Apparent Total Tract Digestibility and Energy Content of Spray-Dried Nannochloropsis oceanica Biomass

The estimates of the TTAD of *N. oceanica* biomass are summarised in Table 6. The TTAD of DM and OM are very similar, whereas those of GE and EE have lower values and those of N are higher. The estimated TTAD of ash was 134.2 ± 6.59%, outside the biologically possible range.

The TTAD estimates for the fatty acids characteristic of *N. oceanica* were consistently high (82.2 ± 0.94% for 16:0, 94.4 ± 0.92% for 16:1c9, 91.7 ± 0.33% for 20:4n-6, and 94.2 ± 0.24% for 20:5n-3). In contrast, the TTAD estimates for fatty acids that were present in low proportions in *N. oceanica* diets but abundant in the basal diet were ill-defined (showing a wide range of confidence limits) and quite a lot lower than in the previous case.

The estimated DE content of *N. oceanica* biomass was 12.7 MJ/kg DM and was obtained using the GE of *N. oceanica* biomass presented in Table 2 and the TTAD of GE herein estimated. The ME content of *N. oceanica* biomass was 12.4 MJ/kg DM and was obtained using the estimated DE content and applying the average ME/DE ratio observed across treatments. Finally, the digestible crude protein (DCP) of the *N. oceanica* biomass was 204 g/kg DM and was obtained using the N of *N. oceanica* biomass presented in Table 2, the N TTAD presented in Table 6, and the conversion factor of 4.78.

### 3.4. Viscosity, pH of Gastrointestinal Contents, and Histomorphology of Small-Intestine Tissues

The information on the viscosity and pH of the gastrointestinal contents of piglets is presented in Table 7. The viscosity of the duodenal and jejunal digesta increased linearly (*p* < 0.001) with *N. oceanica* dietary inclusion. Despite this, no differences between diets were found (*p* > 0.05) for ileum digesta viscosity. Regarding pH readings, no differences were found between diets (*p* > 0.05) for any of the digestive compartments.

Dietary microalga inclusion had no effect on small-intestine histomorphology traits (Table 7), except for the ileum villi lengths that increased linearly (*p* < 0.05).

### 3.5. Volatile Fatty Acid Contents

The VFA contents for caecal and colon digesta are presented in Table 8. Regarding the caecal digesta, the total concentration of VFA (mmol/L) decreased linearly (*p* < 0.001) with *N. oceanica* dietary incorporation. The molar proportion of acetic acid (C2:0) showed a quadratic response (*p* < 0.001), decreasing from the control to 10% *N. oceanica* incorporation and then increasing in the NCO15% group. The opposite occurred for butyric (C4:0) and valeric (C5:0) acids with a quadratic increase (*p* < 0.001) for up to 10% *N. oceanica* incorporation and then a decrease at the highest incorporation level. The molar proportion of iso-butyric acid (iso-C4:0) increased linearly (*p* < 0.001) with dietary *N. oceanica* inclusion, whereas that of propionic acid (C3:0) and iso-valeric acid (iso-C5:0) remained unaltered by the diet.

In the colon digesta, the diets’ effects are less evident. The total concentration of VFA (mmol/L) decreased linearly (*p* = 0.043) with the dietary incorporation of *N. oceanica.* Only C4:0 showed a linear increase in the molar proportion (*p* = 0.048) with the inclusion of *N. oceanica* in the diet.

## 4. Discussion

The nutritional evaluation of algae as a feed ingredient for piglets is limited but available for macroalgae [23]. To the best of our knowledge, this work is the first to estimate the TTAD and energy values of *N. oceanic* for piglet feeding. Indeed, the increasing incorporation of *N. oceanica* in diets at up to 15% in conditions where feed intake was kept similar between treatments allowed us to test the linearity of nutrient TTAD responses. After that, and for those variables that displayed a linear TTAD response, estimating the TTAD of the different microalga biomass nutrients was achieved by linear extrapolation to a theoretical 100% *N. oceanica* diet scenario [24,25]. However, the TTAD estimates obtained for *N. oceanica* biomass should be applied within the range of incorporation level studied (i.e., 0 to 15%). As the incorporation range is wide enough to include most expected practical situations, the TTAD obtained estimates are expected to be suitable for the additive framework of feed formulation. As the experiment was mainly a short-term digestibility trial, the animals were restricted in feeding, resulting, as expected, in a similar feed intake and weight gain among treatments.

In general, the TTAD of diets decreased with the graded incorporation of *N. oceanica* biomass, indicating that diets containing *N. oceanica* have a lower digestibility than the control diet. Despite the small but significant increase in N intake with the graded incorporation of *N. oceanica*, the TTAD of N and N retention efficiency did not differ between treatments. Thus, the metabolic utilisation digestibility of *N. oceanica* N seems to be similar to that of the control diet.

Microalgae present a large content of non-protein N compounds (such as pigments, nucleic acids, and other inorganic compounds) which can lead to an overestimation of CP values when a 6.25 conversion factor is used for its estimation [18]. Based on this, Lourenço et al. [14] analysed 12 species of marine microalgae and proposed an average conversion factor of 4.78, more suitable to microalgae than the factor conventionally used for other feedstuffs. Using this corrected conversion factor is important in order to avoid overestimating the protein content of microalgae biomass. Nevertheless, and as much of the available literature still uses the 6.25 factor to compute microalgae CP contents, we have chosen to present both estimates of the CP of *N. oceanica* biomass in order to enable comparisons.

The digestive function of piglets remained unaltered with the microalgae dietary incorporation, as suggested by intestine histomorphology data and similar growth rates obtained in this study. The TTAD values were lower, and faeces moisture was higher in the second period (week) than in the first period of digestibility measurements. However, these changes cannot be attributed to the dietary inclusion of *N. oceanica* biomass, as no significant interactions between diet and period were detected.

Our estimates indicate that the TTAD for *N. oceanica* DM was 72.5 ± 3.64% and those for OM (71.3 ± 3.52%) and N (71.1 ± 7.59%) were only slightly lower. These values are quite lower than the high-quality protein sources commonly used in diets for weaners such as skimmed milk powder that presents digestibility coefficients for OM, GE, and CP above 92% [26]. Even compared to conventional vegetable protein sources, such as soybean or rapeseed meals, the apparent digestibility determinations of *N. oceanica* DM, OM, and CP are lower. In fact, for growing pigs, the TTAD values are around ≈86% for the OM, GE, and N for soybean meal and 70, 68, and 75% for rapeseed meal, respectively [26]. The DM, OM, and N TTAD of these plant protein sources are negatively related to their NDF content, which increases from zero in skimmed milk powder to 12.4% DM in soybean meal and 28.3% DM in rapeseed meal [26]. However, this pattern should not be applied to the *N. oceanica* biomass due to its low TTAD values and its low NDF content (i.e., 9.2% DM). The analytic determination of NDF in microalgae biomass is very challenging [18], and direct NDF content comparisons with common vegetable sources might not be possible, as it is unclear exactly which specific compounds contribute to the NDF of *N. oceanica* biomass. It is known that *Nannochloropsis* genus cell walls include a cellulosic inner layer covered with an algaenan layer with around 13.5% cell-wall DM [27]. Algaenans are cross-linked long-chain aliphatic hydrocarbon polymers, highly insoluble and practically indigestible. Moreover, due to the immature digestive tract of weaner piglets, their TTAD values are expected to be lower than those obtained for growing pigs [28].

It is often assumed that microalgae cell walls limit the cell contents’ release in the gastrointestinal tract, thus limiting their digestibility [5,13,29]. However, the spray-drying process used to obtain the biomass disrupts the cell wall integrity [30], thus allowing the release and digestion of cell-content components. The high TTAD observed for 20:4n-6 and 20:5n-3, supplied in the diet exclusively by *N. oceanica* biomass, is thus fully consistent with a relatively eased microalgae cell content availability.

Another factor that might contribute to depress microalgae digestibility is the increased intestinal digesta viscosity associated with *N. oceanica* dietary inclusion. Digesta digestibility is mainly determined by the type and amount of polysaccharides present in the diet [31], and it is considered to contribute to the adequate digestion and absorption of nutrients and the correct passage of the contents through the gastrointestinal tract [32]. Despite the lack of information about what would be the expectable range of intestinal digesta viscosity, our team has reported small-intestinal digesta viscosities for piglets fed control diets ranging between 2.6 and 5.9 mPa·s [21,33]. Although the duodenum and jejunum digesta viscosities increased linearly with *N. oceanica* incorporation, all of the observed viscosity values were within the range observed for animals fed control diets. This suggests that the intestinal digesta did not compromise nutrient digestibility in this experiment.

The apparent digestibility of *N. oceanica* biomass EE was relatively low (51.3 ± 8.39% DM), particularly when compared to the OM apparent digestibility (71.3 ± 3.52% DM). This is striking as the apparent digestibility of the main fatty acids in *N. oceanica* biomass ranges from 82.2 to 94.4%. Thus, the low digestibility of EE should be due to the presence of large amounts of compounds with very low digestibility extracted by petroleum ether. Although several compounds might contribute to this low-digestibility EE fraction, chlorophyll, due to either its abundance or low digestibility, is likely the main component responsible for such results. In fact, *Nannochloropsis* spp. might have circa 3.62% DM of chlorophyll α content [34]. We did not measure the chlorophyll α content of the *N. oceanica* biomass used in this experiment. Nonetheless, applying the figure reported by Lee et al. [34], we can estimate that chlorophyll α comprises approximately 19% of the total EE. Moreover, the digestibility of chlorophyll α reported in mammals is very low and ranges between 1 and 3% [35].

The apparent digestibility of fatty acids, particularly unsaturated ones, is consistently above 90%. The TTAD of 16:0 is slightly lower, with values ranging between 82% and 85%. Furthermore, that of 18:0 is notably lower, with values ranging from 3.5 to 20%. Such a response pattern has been previously reported [36,37]. The explanation for the low 18:0 TTAD values is most likely the biohydrogenation of unsaturated C18 FA by the hindgut microbiota as first suggested by Carlson and Bayley [38]. The biohydrogenation of unsaturated fatty acids is well studied in the rumen microbial ecosystem and yields a diversity of trans-octadecenoic intermediates [39]. It is not clear, however, which biohydrogenation pathways occur in the piglet hindgut, but as we detected trans-18:1 isomers peaks in all of the faecal samples analysed, it could be proposed that the biohydrogenation patterns might follow similar pathways as those described for the rumen. Such a proposal warrants however further research.

The recommended diets for post-weaning piglets are quite low in fibre as the quantitative contribution of fermentative activity in the caecum and colon to the whole tract digestion is known to be very low [40]. Despite that, we observed TTAD values for NDF ranging from 66.9% in the control diet to 70% in *N. oceanica*-containing diets. These fairly high TTAD values for NDF are not easily explained and are probably due to methodological and analytical issues. In fact, the quadratic increase in the NDF TTAD with dietary inclusion is inconsistent with the VFA concentration’s linear decrease observed in the caecum and colon and the expected recalcitrant nature of the *N. oceanica* cell wall. The microalgae and the presence of microalgae in the diet impose analytic difficulties in fibre determinations, as already reported in works with seaweeds [41] that coupled to low fibre concentrations, which likely explains most of the inconsistencies found.

The sharp linear increase in the apparent digestibility of ash with the graded dietary inclusion of *N. oceanica* yielded an erroneous estimated TTAD of ash in the microalgae biomass (i.e., 134.2 ± 6.59%). Nevertheless, this indicates that the *N. oceanica* biomass contains a large proportion of the soluble mineral fraction readily available for absorption by piglets. The biomass has a high ash content (i.e., 25.8%) comprised mostly of NaCl, as shown in Table 2, where Na is the mineral with the highest percentage in the biomass (i.e., 5.5%) and furthermore is readily absorbed. Nevertheless, the high mineral content in *N. oceanica* could lead to an electrolyte imbalance, potentially affecting water consumption and leading to negative consequences for animal health [42].

The information obtained in this trial was highly novel and has not been achieved until now. However, it still does not allow for a full characterisation and evaluation of this complex microalga. To complete the nutritional evaluation of *N. oceanica*, the digestibility of its amino acids and its net energy value need to be determined. Such additional information would allow its inclusion in piglets’ diets in a more precise way.

In addition to the lack of nutritional information, another significant challenge associated to the use of this and other microalgae in animal feeding is their production cost, which currently exceeds those of conventional ingredients. These costs are linked to the need for specialised infrastructure, high energy consumption during the phases of biomass production, harvesting, and storage, and the advanced technologies required for biomolecule processing [43,44]. Efforts to enhance the economic viability of microalgae production are being made but considerable progress is still necessary to enable the production of microalgae at a competitive cost comparable to conventional feed ingredients.

## 5. Conclusions

The incorporation of up to 15% of spray-dried *N. oceanica* biomass in the diets of weaner piglets resulted in a linear decrease in the TTAD of DM, OM, EE, and GE but did not affect the dietary N TTAD. No negative effects on the gastrointestinal tract were detected with increasing levels of *N. oceanica* incorporation, although there was an increase in the viscosity of the duodenum and jejunum digesta and a decrease in VFA concentration in the hindgut. The TTAD of nutrients in *N. oceanica* biomass was estimated using the regression approach, allowing for the computation of the DE, ME, and DCP values, which can be used in diet formulation for weaner piglets. In general, *N. oceanica* biomass showed lower TTAD values for DM and CP when compared to conventional protein sources such as soybean meal or whey. Considering that the digestive system of weaner piglets is still developing, caution is needed in the incorporation of *N. oceanica* in piglet nutrition. This study provided novel information about this microalga, characterising it from a nutritional point of view and ascertaining its suitability for piglet nutrition. Further research on the AA content of *N. oceanica* and its specific protein quality utilisation by piglets should be conducted.

## Figures and Tables

**Table 1:** Ingredient composition (% as fed basis), analysed chemical composition (% as DM), gross energy (MJ/kg DM), and fatty acid composition (% of total FA) of the four experimental diets.

	Diets ^1^			
	Control	NCO5%	NCO10%	NCO15%
**Ingredient (% as fed basis)**				
Wheat	43.7	41.5	39.0	37.0
Corn	15.0	14.2	13.5	12.7
Soybean meal	25.0	23.7	22.5	21.0
Dry milk	10.0	9.5	9.0	8.5
Vegetable oil	3.0	2.8	2.7	2.5
*Nannochloropsis oceanica*	0.0	5.0	10.0	15.0
L-Lysine	0.5	0.5	0.5	0.5
DL-Methionine	0.1	0.1	0.1	0.1
Threonine	0.1	0.1	0.1	0.1
Calcium carbonate	0.5	0.5	0.5	0.5
Dicalcium phosphate	1.3	1.3	1.3	1.3
Sodium chloride	0.3	0.3	0.3	0.3
Mineral and vitamin complex ^2^	0.5	0.5	0.5	0.5
**Chemical composition (% DM)**				
Dry matter (%)	90.5	90.6	90.1	90.9
Ash	5.8	6.9	8.0	9.1
Crude protein (N × 6.25)	20.6	21.6	21.4	22.6
Crude protein adjusted ^3^	20.6	20.8	21.0	21.2
Ether extract	6.0	6.7	7.2	7.9
NDF ^4^	10.8	11.2	11.8	12.4
ADF	3.6	3.6	3.4	3.4
Lignin	0.8	0.8	0.8	0.8
Gross energy (MJ/kg DM)	18.57	18.43	18.53	18.62
**Fatty acid composition (% total FA)**				
12:0	0.4	0.4	0.4	0.4
14:0	2.4	1.4	1.3	1.2
15:0	0.03	0.1	0.1	0.1
16:0	11.0	13.9	16.5	18.7
16:1c9	0.1	3.5	6.8	9.6
17:0	0.1	0.2	0.1	0.1
18:0	3.2	2.9	2.7	2.5
18:1c9	23.6	22.6	20.6	18.2
18:1c11	0.8	0.7	0.7	0.7
18:2n-6	55.5	49.6	43.8	39.6
20:0	0.2	0.2	0.2	0.2
18:3n-3	2.3	1.7	1.5	1.3
20:2n-6	0.5	0.4	0.4	0.3
20:4n-6	0	0.7	1.3	1.8
20:5n-3	0	1.8	3.6	5.3
Total FA (mg/g MS)	54.77	58.96	63.38	65.97

Abbreviations: DM = dry matter; NDF = neutral detergent fibre; ADF = acid detergent fibre. ^1^ Control = diet without *N. oceanica*; NCO5% = 95% control diet and 5% *N. oceanica*; NCO10% = 90% control diet and 10% *N. oceanica*; NCO15% = 85% control diet and 15% *N. oceanica*. ^2^ Mineral and vitamin mixture supplied per kg of diet: 6500 UI of vitamin A; 1500 UI of vitamin D_3_; 15.0 mg of vitamin E; 1.0 mg of vitamin K_3_; 1.0 mg of vitamin B_1_; 3.0 mg of vitamin B_2_; 6.0 mg of vitamin B_6_; 0.02 mg of vitamin B_12_; 10.0 mg of pantothenic acid; 15.0 mg of nicotinic acid; 0.5 mg of folic acid; 0.03 mg of biotin; 115.0 mg of betaine; 20.0 mg of vitamin C; 100.0 mg of copper; 100 mg of iron; 0.5 mg of iodine; 50.0 mg of manganese; 0.15 mg of selenium; 100 mg of zinc; 3 mg of butylated hydroxytoluene. ^3^ Calculated considering the 4.78 conversion factor for the contribution of the microalgae N [14] and 6.25 for all of the other ingredients. ^4^ NDF assayed with heat-stable amylase and expressed inclusive of residual ash.

**Table 2:** *Nannochloropsis oceanica* chemical composition (% DM), gross energy (MJ/kg DM), and fatty acid composition (% total FA).

	*Nannochloropsis oceanica*
**Chemical composition (% DM)**	
Dry matter (%)	96.1
Ash	25.8
Organic matter	74.2
N	5.2
Crude protein (N × 6.25)	32.5
Crude protein (N × 4.78) ^1^	24.9
Ether extract	19.4
NDF ^2^	9.2
Gross energy (MJ/kg DM)	19.79
**Fatty acid composition (% total FA)**	
14:0	4.4
16:0	20.0
16:1c9	26.5
18:0	0.4
18:1c9	3.9
18:2n-6	3.3
18:3n-3	0.8
20:0	0.1
20:4n-6	7.5
20:5n-3	28.9
Total FA (mg/g DM)	120
**Mineral content (% DM)**	
Ca	0.5
P	0.9
K	2.7
Na	5.5
S	0.6
Mg	0.8
Cu	0.001
Fe	0.06
Mn	0.01
Zn	0.003

Abbreviations: DM = dry matter; NDF = neutral detergent fibre; Ca = calcium; P = phosphorus; K = potassium; Na = sodium; S = sulphur; Mg = magnesium; Cu = copper; Fe = iron; Mn = manganese; Zn = zinc. ^1^ Conversion factor for microalgae [14]. ^2^ NDF assayed with heat-stable amylase and expressed exclusive of residual ash.

**Table 3:** Effect of diets on feed intake, growth parameters of piglets, and faecal dry matter content.

	Diets ^1^				SEM	*p*-Values ^2^	
	Control	NCO5%	NCO10%	NCO15%		L	Q
Initial weight (kg)	15.6	15.4	15.6	15.5	0.29	0.945	0.955
Final weight (kg)	23.6	22.8	23.4	23.1	0.33	0.764	0.706
DM intake (g/d)	851	822	868	832	8.2	0.883	0.836
ADG (g)	571	529	555	545	14.0	0.694	0.592
Faecal DM (g/kg)	298	312	284	271	20.2	0.237	0.502

Abbreviations: ADG = average daily gain; DM = dry matter. ^1^ Control = diet without *N. oceanica*; NCO5% = 95% control diet and 5% *N. oceanica*; NCO10% = 90% control diet and 10% *N. oceanica*; NCO15% = 85% control diet and 15% *N. oceanica*. ^2^ *p*-values for L = linear polynomial contrast, and Q = quadratic polynomial contrast.

**Table 4:** Effect of dietary inclusion of *Nannochloropsis oceanica* on diets’ total tract apparent digestibility.

	Diets ^1^				SEM	*p*-Values ^2^	
	Control	NCO5%	NCO10%	NCO15%		L	Q
**TTAD (%)**							
Dry matter	89.3	88.0	87.6	86.6	0.45	<0.001	0.786
Ash	66.4	69.7	73.9	76.3	0.82	<0.001	0.584
Organic matter	90.7	89.4	88.8	87.7	0.44	<0.001	0.870
N	84.0	83.1	82.5	82.1	0.95	0.149	0.768
Ether extract	84.9	82.0	80.5	79.9	1.02	0.002	0.300
NDF	66.9	69.4	72.0	70.2	0.92	0.007	<0.001
ADF	55.7	58.0	55.1	49.1	1.65	0.005	0.020
Gross energy	89.3	87.6	86.7	85.5	0.50	<0.001	0.620
Fatty acids							
16:0	85.0	82.1	82.1	82.7	1.63	0.372	0.301
16:1c9	90.7	95.0	94.1	94.2	1.59	0.195	0.219
18:1c9	98.2	97.6	96.8	96.7	0.42	0.008	0.551
18:2n-6	99.0	98.4	97.7	97.9	0.37	0.024	0.308
18:3n-3	98.1	96.3	95.1	94.4	0.50	<0.001	0.302
20:4n-6	-	91.9	91.2	92.0	0.57	0.854	0.284
20:5n-3	-	94.5	93.7	94.4	0.41	0.876	0.159

Abbreviations: TTDA = total tract apparent digestibility; NDF = neutral detergent fibre; ADF = acid detergent fibre. ^1^ Control = diet without *N. oceanica*; NCO5% = 95% control diet and 5% *N. oceanica*; NCO10% = 90% control diet and 10% *N. oceanica*; NCO15% = 85% control diet and 15% *N. oceanica*. ^2^ *p*-values for L = linear polynomial contrast, and Q = quadratic polynomial contrast.

**Table 5:** Effect of dietary inclusion of *Nannochloropsis oceanica* on the nitrogen balance of piglets and the digestible and metabolisable energy content of their diets.

	Diets ^1^				SEM	*p*-Values ^2^	
	Control	NCO5%	NCO10%	NCO15%		L	Q
**Ingested N (g/d)**	25.5	25.8	27.2	27.3	0.49	0.005	0.923
**Retained N (g/d)**	16.3	16.4	17.1	18.1	0.68	0.058	0.498
**N Retention efficiencies**							
NRC ^3^	76.0	76.1	75.8	80.5	2.04	0.165	0.266
NPRC ^4^	63.9	63.3	62.9	66.2	1.90	0.462	0.312
**Energy values of diets (MJ/kg DM)**							
DE	17.67	17.32	17.15	16.91	0.100	<0.001	0.620
ME	17.26	16.93	16.76	16.52	0.097	<0.001	0.620
ME/DE	0.975	0.974	0.965	0.979	0.0031	0.867	0.034

Abbreviations: DE = digestible energy; ME = metabolisable energy. ^1^ Control = diet without *N. oceanica*; NCO5% = 95% control diet and 5% *N. oceanica*; NCO10% = 90% control diet and 10% *N. oceanica*; NCO15% = 85% control diet and 15% *N. oceanica*. ^2^ *p*-values for L = linear polynomial contrast, and Q = quadratic polynomial contrast. ^3^ NRC = nitrogen retention coefficient = (retained N/absorbed N) × 1000. ^4^ NPRC = nitrogen practical retention coefficient = (retained N/ingested N) × 1000.

**Table 6:** Prediction of total tract apparent digestibility (TTAD) and energy values of Nannochloropsis oceanica biomass incorporated into the diets using the regression method.

	Estimated Values ± SE	Confidence Limits
**TTAD (%)**		
Dry matter ^1^	72.5 ± 3.64	64.9 ↔ 80.0
Organic matter ^1^	71.3 ± 3.52	64.0 ↔ 78.6
N ^2^	82.9 ± 0.47	81.9 ↔ 83.9
Ether extract ^1^	51.3 ± 8.39	34.1 ↔ 68.6
Gross energy (GE) ^1^	64.4 ± 4.03	56.0 ↔ 72.1
Fatty acids		
16:0 ^2^	82.2 ± 0.94	80.3 ↔ 84.3
16:1c9 ^2^	94.4 ± 0.92	92.5 ↔ 96.3
20:4 n-6 ^2^	91.7 ± 0.33	90.9 ↔ 92.4
20:5 n-3 ^2^	94.2 ± 2.4	93.6 ↔ 94.7
**Energy content (MJ/kg DM)**		
Digestible energy (DE) ^3^	12.74	-
Metabolisable energy (ME) ^4^	12.40	-
Digestible CP (DCP), % DM ^5^	20.4	-

Abbreviations: TTDA = total tract apparent digestibility; DM = dry matter. ^1^ Estimates of the standard errors and confident limits obtained from regression equations of the incorporation of *N. oceanica* on TTAD solved to 100%. ^2^ When no linear effect of the dietary incorporation of *N. oceanica* was observed, the estimates of the standard errors and confident limits were obtained from the common LSMeans estimate of the TTAD values of the treatments containing *N. oceanica.* ^3^ DE (MJ/kg DM) = GE (Table 2) × GE_TTAD. ^4^ ME (MJ/kg DM) = DE × ME/DE, where ME/DE = 0.972 ± 0.028. ^5^ DCP = (N × N_TTAD) × 4.78.

**Table 7:** Effect of dietary Nannochloropsis oceanica inclusion on viscosity and pH of gastrointestinal contents and intestinal histomorphology of the small intestine of piglets.

	Diets ^1^				SEM	*p*-Values ^2^	
	Control	NCO5%	NCO10%	NCO15%		L	Q
**Viscosity (** **mPa·s)**							
Duodenum + Jejunum	3.22	4.04	4.35	4.78	0.160	<0.001	0.433
Ileum	4.88	4.90	4.66	4.85	0.090	0.706	0.673
**pH**							
Stomach	4.13	3.78	4.36	4.12	0.161	0.715	0.861
Duodenum + Jejunum	5.47	5.75	5.60	5.39	0.083	0.593	0.148
Ileum	6.67	6.50	6.42	6.49	0.066	0.320	0.403
Caecum	5.93	6.12	5.84	5.78	0.068	0.249	0.359
Colon	6.10	6.29	6.23	6.05	0.077	0.752	0.252
**Intestinal histomorphology**							
**Villi length (** **μm** **)**							
Duodenum	430	500	463	443	16.8	0.988	0.205
Jejunum	390	491	420	485	25.7	0.360	0.726
Ileum	330	355	378	418	15.4	0.043	0.801
**Villi width (μm)**							
Duodenum	170	195	175	207	5.9	0.075	0.755
Jejunum	133	144	150	148	3.6	0.119	0.364
Ileum	147	168	161	171	4.4	0.102	0.510
**Crypts depth (μm)**							
Duodenum	485	569	509	554	22.2	0.468	0.674
Jejunum	353	380	423	355	14.8	0.709	0.119
Ileum	354	323	367	355	12.1	0.668	0.699
**Villi/crypts ^3^**							
Duodenum	0.89	0.89	0.96	0.83	0.040	0.746	0.449
Jejunum	1.10	1.30	1.04	1.42	0.080	0.322	0.535
Ileum	0.94	1.12	1.10	1.22	0.070	1.199	0.848

^1^ Control = diet without *N. oceanica*; NCO5% = 95% control diet and 5% *N. oceanica*; NCO10% = 90% control diet and 10% *N. oceanica*; NCO15% = 85% control diet and 15% *N. oceanica*. ^2^ *p*-values for L = linear polynomial contrast, and Q = quadratic polynomial contrast. ^3^ Height of villi/depth of crypts.

**Table 8:** Effect of dietary Nannochloropsis oceanica inclusion on volatile fatty acids profile in the caecum and colon digesta of piglets.

	Diets ^1^				SEM	*p*-Values ^2^	
	Control	NCO5%	NCO10%	NCO15%		L	Q
**Caecum**							
**Total VFA (mmol/L)**	22.3	13.7	14.4	12.3	1.12	<0.001	0.067
**VFA profile (mmol%)**							
C2:0	56.1	44.3	39.3	45.9	1.58	<0.001	0.001
C3:0	26.2	26.1	23.9	24.9	0.80	0.419	0.736
C4:0	13.0	21.1	26.2	21.4	1.28	<0.001	0.001
C5:0	2.98	3.87	6.88	3.17	0.45	0.232	0.002
iso-C4:0	1.39	3.49	2.64	3.79	0.24	<0.001	0.151
iso-C5:0	0.40	1.14	1.12	0.88	0.12	0.180	0.046
**Colon**							
**Total VFA (mmol/L)**	25.3	19.5	21.1	19.4	0.94	0.043	0.246
**VFA profile (mmol%)**							
C2:0	53.7	59.5	52.8	53.4	1.19	0.455	0.258
C3:0	24.8	20.7	22.1	23.2	0.69	0.546	0.066
C4:0	14.5	13.3	16.9	17.3	0.70	0.048	0.560
C5:0	3.80	2.93	4.50	3.07	0.27	0.778	0.591
iso-C4:0	2.27	2.10	2.29	2.05	0.14	0.725	0.917
iso-C5:0	1.00	1.43	1.38	1.05	0.14	0.935	0.206

Abbreviations: VFA = volatile fatty acids. ^1^ Control = diet without *N. oceanica*; NCO5% = 95% control diet and 5% *N. oceanica*; NCO10% = 90% control diet and 10% *N. oceanica*; NCO15% = 85% control diet and 15% *N. oceanica*. ^2^ *p*-values for L = linear polynomial contrast, and Q = quadratic polynomial contrast.

## Data Availability

The original contributions presented in the study are included in the article. Further inquiries can be directed to the corresponding authors.

## Supplementary Materials

The following supporting information can be downloaded at https://www.mdpi.com/article/10.3390/ani14243575/s1, Table S1: Least square means and *p*-values for the period effect and period × diet interaction of the variables evaluated in the week 1 and week 2 periods of the experiment.
