# Supplementary material for: Nutritional Value of Nannochloropsis oceanica for Weaner Piglets
Source: Animals (Basel). 2024 Dec 11;14(24):3575. doi: 10.3390/ani14243575 (PMC11672712; doi:10.3390/ani14243575)
Supplement: Supplementary file 1 [file animals-14-03575-s001.zip › animals-3327113-supplementary.pdf]

Table S1. Least square means and *P* values for the period effect and period × diet interaction of the variables evaluated in the week 1 and week 2 periods of the experiment.

	Period		SEM	*p*-Value	
	**Week1**	**Week2**		**Period**	**Wk×D**
**DMI (g/d)**	819	867	8.5	0.004	0.290
**ADG (g/d)**	647	453	19.6	<0.001	0.615
**TTAD (%)**					
Dry matter	89.0	86.8	0.29	<0.001	0.416
Ash	73.5	69.6	0.58	0.001	0.666
Organic matter	90.2	88.1	0.28	<0.001	0.409
N	85.3	80.5	0.56	<0.001	0.631
Ether extract	83.8	79.8	0.66	<0.001	0.836
NDF	71.6	67.6	0.61	<0.001	0.622
ADF	57.3	51.7	1.06	<0.001	0.116
Gross energy	88.6	85.9	0.33	<0.001	0.492
Total fatty acid	93.6	90.1	0.48	<0.001	0.285
14:0	96.0	84.6	0.56	<0.001	<0.001
16:0	86.1	79.8	0.98	<0.001	0.068
16:1c9	95.8	88.0	1.04	0.002	0.094
18:0	25.1	-0.136	7.60	0.005	0.492
18:1c9	95.8	91.2	0.26	<0.001	0.342
18:2 n-6	99.1	97.5	0.23	<0.001	0.314
18:3 n-3	97.4	94.5	0.35	<0.001	0.345
20:4 n-6	95.2	88.1	0.47	<0.001	0.597
20:5 n-3	97.3	91.1	0.29	<0.001	0.301
**Faecal DM (g/kg)**	322	261	10.1	<0.001	0.564
**Ingested N (g/d)**	25.7	27.2	0.33	0.004	0.293
**Retained N (g/d)**	17.2	16.7	0.47	0.450	0.348
**(N retained/N absorbed)×1000**	78.3	76.0	1.33	0.196	0.475
**(N retained/N ingested)×1000**	66.9	61.3	1.26	0.003	0.554
**DE (MJ/kg DM)**	17.54	16.99	0.065	<0.001	0.492
**ME (MJ/kg DM)**	17.13	16.60	0.064	<0.001	0.492
**ME/DE**	0.974	0.972	0.002	0.573	0.649

Abbreviations: DMI = Dry matter intake; ADG = Average daily gain; TTAD = Total tract apparent digestibility; NDF = Neutral detergent fibre; ADF = Acid detergent fibre; DE = Digestible energy; ME = Metabolisable energy; DM = Dry matter.
